# A Novel Phage PD-6A3, and Its Endolysin Ply6A3, With Extended Lytic Activity Against *Acinetobacter baumannii*

**DOI:** 10.3389/fmicb.2018.03302

**Published:** 2019-01-09

**Authors:** Minle Wu, Kongying Hu, Youhua Xie, Yili Liu, Di Mu, Huimin Guo, Zhifan Zhang, Yingcong Zhang, Dong Chang, Yi Shi

**Affiliations:** ^1^Department of Clinical Laboratory, Shanghai Pudong Hospital, Fudan University, Shanghai, China; ^2^Key Laboratory of Medical Molecular Virology, School of Basic Medical Sciences, Fudan University, Shanghai, China; ^3^Department of Clinical Laboratory, Shanghai Public Health Clinical Center, Shanghai, China; ^4^Department of Clinical Laboratory, Shanghai Fourth People’s Hospital Affiliated to Tongji University School of Medicine, Shanghai, China

**Keywords:** multidrug resistance *Acinetobacter baumannii*, endolysin, ESKAPE, sepsis, phage therapy, mice model

## Abstract

With widespread abuse of antibiotics, bacterial resistance has increasingly become a serious threat. *Acinetobacter baumannii* has emerged as one of the most important hospital-acquired pathogens worldwide. Bacteriophages (also called “phages”) could be used as a potential alternative therapy to meet the challenges posed by such pathogens. Endolysins from phages have also been attracting increasing interest as potential antimicrobial agents. Here, we isolated 14 phages against *A. baumannii*, determined the lytic spectrum of each phage, and selected one with a relatively broad host range, named vB_AbaP_PD-6A3 (PD-6A3 for short), for its biological characteristics. We over-expressed and purified the endolysin (Ply6A3) from this phage and tested its biological characteristics. The PD-6A3 is a novel phage, which can kill 32.4% (179/552) of clinical multidrug resistant *A. baumannii* (MDRAB) isolates. Interestingly, *in vitro*, this endolysin could not only inhibit *A. baumannii*, but also that of other strains, such as *Escherichia coli* and methicillin-resistant *Staphylococcus aureus* (MRSA). We found that lethal *A. baumannii* sepsis mice could be effectively rescued *in vivo* by phage PD-6A3 and endolysin Ply6A3 intraperitoneal injection. These characteristics reveal the promising potential of phage PD-6A3 and endolysin Ply6A3 as attractive candidates for the control of *A. baumannii*-associated nosocomial infections.

## Introduction

*Acinetobacter baumannii* is a non-fermentative, Gram-negative bacillus that can be universally found in nature. It is a conditional pathogen that has been proven to be one of the major pathogens causing hospital-acquired infections, such as bacteremia, urinary tract infections, and ventilator-associated pneumonia (Doi et al., 2015).

An increasing number of *A. baumannii* strains have now been clinically proven to be multi-drug, and even pan-drug resistant (Lin, 2014; Shivaswamy et al., 2015; Khakhum et al., 2016). According to CHINET surveillance in China, *A. baumannii* accounts for 10.77% of all clinical isolates, and is ranked the third most prevalent (Hu et al., 2016). The majority of these isolates are multidrug resistant *A. baumannii* (MDRAB), which is resistant to nearly all accessible antimicrobials. Therefore, the development of new tools to effectively manage *A. baumannii* infections is of great importance.

Bacteriophages (also called phages), as natural enemies of bacteria, exist abundantly in environments shared by their hosts. Phages were first discovered by Felix d’Hérelle in 1917 (Billiau, 2016; El-Shibiny and El-Sahhar, 2017). The lytic phages can specifically lyse pathogenic bacteria within their host range. In recent years, a huge importance has been given to the phage therapy and phage have been proposed as a valuable approach to control multiple pathogens (Melo et al., 2018).

In addition, phage endolysins, which phages use to digest the host bacterial cell wall to release progeny phages, have been suggested to be potent antibacterial agents in the treatment of Gram-positive pathogens both *in vitro* and in animal models (Yang et al., 2015; Zhang et al., 2016). However, the Gram-negative bacteria prevent the access of natural endolysins to peptidoglycan layer because of their outer membranes, thus making the exogenously added endolysins limited (Lim et al., 2014). Therefore, endolysins with strong lytic ability for the treatment of Gram-negative bacteria especially *A. baumannii* are urgently required.

We isolated and characterized a novel *A. baumannii* phage, PD6A3, and over-expressed its endolysin Ply6A3. Furthermore, the characteristics of the novel endolysin were evaluated. We then applied phage PD6A3 and endolysin Ply6A3 to the treatment of septic infections in mice. The present results support the potential use of phage PD6A3 and endolysin Ply6A3 as alternative antibacterial agents against drug-resistant *A. baumannii* infections.

## Materials and Methods

### Bacterial Strains and Culture Conditions

The clinical bacterial strains, human cells, plasmids, and primers used in the present study are listed in Table 1. Altogether, 552 clinical MDRAB isolates (Supplementary Table 1), and *Pseudomonas aeruginosa*, *Escherichia coli*, *Klebsiella pneumonia, Staphylococcus aureus*, and *Enterococcus faecium*, each comprising 40 isolates were collected between 2014 and 2017. Theses isolates were all collected from different patients at seven different hospitals in Shanghai, China and we have gotten written informed consents from these patients. Isolates were identified using the Vitek 2 compact system (Biomerieux, France). Antimicrobial susceptibility testing was performed using the broth microdilution method, recommended by the Clinical and Laboratory Standards Institute (CLSI, 2017). All isolates were identified by 16S rDNA sequencing analysis. The *E. coli* DH5α and *E. coli* BL21 (DE3) cells were purchased from TransGen Biotech, China. The overnight bacterial culture was 1:100 inoculated to fresh Luria-Bertani (LB) broth and cells were grown at 37°C with shaking at 220 rpm (Yang et al., 2015).

**Table 1:** Primers, phage, bacterial strains, and plasmids used in this study.

Name	Characteristics	Source
Forward primer	CGCTCGAGATGATTCT GACTAAAGACGGATTTAG	Sangon Biotech
Reverse primer	GCGAAGCTTCTATA AGCTCCGTAGAGCG	Sangon Biotech
pCold-SUMO	Expression vector (kanamycin resistant)	Our Laboratory Collection
pCold-SUMO-Ply6A3	Recombinant vector (kanamycin resistant)	This study
DH5α *E. coli*	Amplification host for plasmid	Transgen Biotech
BL21(DE3) *E. coli*	Expression host for recombinant plasmid	Transgen Biotech
PD6A3	*A. baumannii* phage	Our Laboratory Collection
Ply6A3	The protein expressed by the endolysin gene	This study
AB1-AB200	MDR-*A. baumannii*, 200 clinical strains	Our Laboratory Collection
AB32	One of the host bacteria of Phage PD6A3	Our Laboratory Collection
AB01-AB040	PDR-*A. baumannii*, 40 clinical strains	Our Laboratory Collection
*Enterococcus faecium*	Vancomycin resistant, 40 clinical strains	Our Laboratory Collection
*Staphylococcus aureus*	Methicillin resistant, 40 clinical strains	Our Laboratory Collection
MRSA6	One of the host bacteria of Phage PD6A3	Our Laboratory Collection
*Klebsiella pneumonia*	Extended spectrumβ lactamases, 40 clinical strains	Our Laboratory Collection
*Pseudomonas aeruginosa*	Multi-drug Resistance, 40 clinical strains	Our Laboratory Collection
*Escherichia coli*	Extended spectrumβ lactamases, 40 clinical strains	Our Laboratory Collection
HEK293T cells	Human renal epithelial cells	Our Laboratory Collection
THP-1 cells	Human mononuclear macrophage	Our Laboratory Collection

*PD6A3, a novel *Acinetobacter baumannii* Phage. Ply6A3, the endolysin encoded by a novel *Acinetobacter baumannii* Phage *PD6A3* gene. AB32, the No. 32 strains of multi-drug resistant *Acinetobacter baumannii*. MRSA6, the No. 6 strains of methicillin-resistant *Staphylococcus aureus*. HEK293T cells, human embryonic kidney cells 293. THP-1 cells, a human monocytic cell line derived from an acute monocytic leukemia patient*.

### Phage Isolation and Lytic Spectrum of Single Phages and Phage Cocktail

In this study, 27 non-duplicate clinical strains of extensively drug-resistant (XDR) *A. baumannii* were used as indicators of phage isolation. All phages were isolated from sewage (Yele et al., 2012). The supernatant (20 mL) from the sewage was added to 10 mL 3 × LB broth and 5 mL of mixed *A. baumannii* culture, then incubated at 37°C and 100 rpm/min overnight. We used the double-layer agar method to incubate and purify the supernatants which including the phages. Each individual phage was purified using several consecutive rounds of plaque picking until single-plaque morphology was observed. Eventually, 14 virulent phages were obtained. To enrich the phages, each phage was mixed with its host cells by certain proportion then grown in LB soft agar overlays (0.75% agar) overnight. Then the lysate was collected in SM buffer, enriched at 4°C overnight using polyethylene glycol (PEG) 8000, further purified with CsCl density gradient ultracentrifugation and stored at 4°C (Hua et al., 2018).

Then, one drop of phage PD6A3 solution was added onto a copper mesh grid, after natural precipitation about 10 min, 50 μl 2% phosphotungstic acid was added to the grid. After drying, we used a Hitachi transmission electron microscope H-9500 (Japan) to examine the morphology of the phage (Huang et al., 2013).

Phage titers were assessed using double-layer agar method and represented by plaque-forming unit. The lytic spectrum of each phage was tested using the spot assay (Huang et al., 2013; Kitti et al., 2014). We also tested the lytic spectrum of a phage cocktail, which contained all 14 phages.

### Biological Characterization and Lytic Effect of Phage PD-6A3

Thermal and pH stability tests were carried out using the double-layer technique (Capra et al., 2004). In the thermal stability tests, aliquots of phage PD6A3 preparations (10^9^ CFU/ml) were treated at pH 7 and at different temperatures (4, 37, 40, 50, 60, and 70°C) in SM Buffer for 1 h. As for the pH stability experiments, phage preparations were treated with various pH buffers at (from pH 2 to pH 11) at 37°C in SM Buffer for 1 h and the phage titers were assessed. An adsorption experiment was performed according to Adams (Adams, 1959). Briefly, exponentially grown AB32 (a clinical pan-drug resistant *A. baumannii*) were infected with the phage at the MOI of 0.1. Aliquots of 1 ml were taken every minute (from 1 to 10 min) and then the supernatant containing the non-adsorbed phages were titrated using the double-layer method. A one-step growth experiment was determined using the method described by Pajunen et al. (2000). The AB32 was infected by Phage PD6A3 at a MOI of 0.01 and adsorbed for 5 min. During the incubation, samples were taken periodically (10-min intervals, over a period of 50 min) and immediately assayed for plaque titer. In the assay of phage infection curve, AB32 were mixed with the phage PD6A3 at different MOIs (from 0.00001 to 1) at 37°C. The control experiments were performed using equal volume of SM buffer. The OD600 values were measured at 30-min intervals until 7 h post-infection.

### Extraction, Sequencing, and Bioinformatics of Phage Genomic DNA

The phage PD6A3 was purified by CsCl gradient ultracentrifugation method as described before. Genome sequence of the phage was obtained using the Illumina HiSeq 3000 Sequencing platform (Illumina, San Diego, CA, United States) and the SOAPdenovo2 software for sequence assembly. Prediction and annotation between genes from phage PD6A3 and other phages were performed using GeneMarkS and Blast^1^ (Jin et al., 2012). Functional annotation of ORFs and homology assignments between genes from phage PD6A3 and other phages were performed by the BLASTp^1^. Expasy compute PI/MV tool^2^ was used to determine isoelectric pH and molecular weight of the predicted ORFs. The Easyfig software was used to conducted the alignment of functional proteins of phage PD6A3 (Hua et al., 2018). An alignment of amino acid sequences between endolysin Ply6A3 and other endolysin gene was created using the Clustal X2 program (Kong and Ryu, 2015). The whole genome of phage PD6A3 was deposited at GenBank under accession number KY388102.1.

### Identification and Cloning of the Putative Endolysin From Phage PD-6A3

Gene prediction was performed as described above. The phage PD-6A3 endolysin encoding gene was amplified with P1 and P2 primers (Table 1) and PrimeSTAR HS DNA polymerase kits (TaKaRa, China). The PCR products were purified using a Rapid Mini Plasmid Kit (TIANGEN, China). Furthermore, PCR products were resolved in a 1% agarose gel before purified. The gene was then linked to an expression vector named pCold-SUMO, which comprised a 6× His tag and SUMO tag (Guo et al., 2016). The recombinant vector was transformed into *E. coli* DH5α cells. After confirmation by restriction enzyme digestion and sequence analysis, the correct plasmids were transformed into the BL21(DE3) cells for over-expression.

### Over-Expression and Purification of Endolysin Ply6A3

The BL21(DE3) cells were grown in LB medium containing kanamycin (50 μg/mL, Sangon Biotech, China) at 37°C overnight, diluted 1:100 in 0.5 L LB medium, then incubated until the OD 600 reached 0.6∼0.8. Isopropyl β-D-thiogalactoside (IPTG) (TIANGEN, China) was added until the final concentration was 1 mM, then the mixture was incubated at 16°C for 18 h at 120 rpm. The following procedures were all performed in an ice-cold water bath (Diez-Martinez et al., 2015). The precipitate was resuspended in 25 mL PBS buffer (pH 7.4). The cell lysate was disrupted by sonication (Cole-Parmer, Ultrasonic Processors) (10 s pulse, 20 s rest over 30 min) before centrifuged at 12,000 × *g* 4°C (Lai et al., 2011). The His-SUMO-Ply6A3 was purified using a Ni-nitrilotriacetic acid column (Novagen, United States), and equilibrated with a binding buffer (20 mM Tris-HCl, 300 mM NaCl, 10 mM imidazole; pH 8.0) at room temperature. The supernatant was filtered using a 0.22-mm membrane, after it was passed through the column at least three times. The His-SUMO- ply6A3 in the supernatant was washed with binding buffer (20 mM Tris-HCl, 300 mM NaCl, 10 mM imidazole; pH 8.0) three times, and the same washing buffer was then eluted with elution buffer (20 mM Tris-HCl, 300 mM NaCl, 50 mM imidazole; pH 8.0). All samples were stored for SDS-PAGE and western blot analyses.

### Cleavage of the SUMO Tag and Protein Concentration

The reaction assay was performed in a dialysis bag in a 4°C-refrigerator overnight. The SUMO protease and purified His-SUMO-ply6A3 were placed in dialysis at a ratio of 50:1 (vol/vol). The His-SUMO protein was cleaved with continuous stirring overnight, and the dialysis bag was placed in PBS buffer (pH 7.0) (Guo et al., 2016). The enzyme digested product was then purified using an affinity chromatography column (Novagen. United States). The protein solution was collected into a protein concentration tube with a molecular diameter of 10 kD (Amicon Ultra), and centrifuged at 4,000 × *g* at 4°C for 30 min. The concentration of the purified protein was then measured using a Micro BCA^TM^ protein assay kit (Tiangen, China).

### SDS-PAGE and Western Blot Analysis

The samples collected during expression, purification, and cleavage were subjected to 12% SDS-PAGE, and then transferred to polyvinylidene fluoride (PVDF) membranes (Kong and Ryu, 2015). The PVDF membranes were blocked with 5% bovine serum albumin in phosphate buffered saline with Tween 20 (PBST) for 3 h and then incubated with anti-His antibody (1:3000) (Sangon Biotech, China) overnight, followed by peroxidase-affiniPure goat anti-mouse IgG (1:10000) (Sangon Biotech, China) for 1 h.

### Detection of Peptidoglycan Degradation by PlyAB3 Using the Diffusion Method

According to the enrichment technique recommended by Sun et al. (2005). The *AB32* was incubated in 0.5 L LB medium at 37°C for 24 h then harvested by centrifugation, before washed several times with ddH_2_O. Then placed in a boiling water bath for 45 min and broken by sonication. Next, centrifuged at 1000 r/min for 10 min, the supernatant was decanted. Centrifuged the supernatant again, the precipitate was incubated in 4% SDS before washed with ddH_2_O and dehydrated alcohol, digested with 0.02% trypsin in PBS (pH 7.5) for 24 h. The walls were resuspended in 10% trichloroacetic acid at 70°C for 30 min. Then harvested by centrifugation and washed by ddH_2_O, the peptidoglycan was then mixed in proportion (0.5%) with 15% SDS-polyacrylamide gel. After solidification, the gel was perforated with a sterilizing puncher. A volume of 20 μL endolysin Ply6A3 (2 mg/mL) was added to the hole formed. The negative control was PBS, and the positive control was lysozyme (2 mg/mL). The mixtures were sealed in a wet box and cultured at 37°C overnight, following which the samples were dyed with methylene blue stain for 2 h, and bleached to transparent strips with ddH_2_O (Blázquez et al., 2016).

### Cytotoxicity of Phage PD-6A3 and Endolysin Ply6A3 to HEK293T and THP-1 Cells

The human embryonic kidney cells 293 (HEK293T) and a human monocytic cell THP-1 cells were both supplemented with fetal bovine serum (10%) and incubated in 5% CO_2_ at 37°C (Shen et al., 2018). Cells were seeded into 96-well plates (10^4^ cells/well), then incubated for 24 h. They were then incubated with different concentrations of phage PD-6A3 or endolysin Ply6A3 for an additional 8 and 24 h (Yin et al., 2018). Cell viability was measured using the Cell Counting Kit-8 (CCK-8; Dojindo, Kumamoto, Japan) and absorbance was measured at 450 nm.

### Biological Characterization and Enzyme Activity of Endolysin Ply6A3

To test the thermal and pH stability of endolysin Ply6A3, host bacteria Ab32, which grew to log phase, was repeatedly suspended three times with PBS. A volume of 100 μL endolysin (1 mg/mL, dissolved in PBS) was combined with 100 μL Ab32 (1 × 10^9^ cfu/mL), and added to a 96-well plate incubated at temperatures of 22, 27, 32, 37, and 42°C, at pH 7 for 0.5 h. For the pH assay, the mixture was incubated at pH 5.5, 6.0, 6.5, 7.0, 7.5, 8.0, and 8.5, at 37°C for 0.5 h (Kong and Ryu, 2015). A mixture of bacteria and PBS was used as the blank control group. Absorbance was measured before and after incubation. Based on the two readings, the percentage drop in bacterial turbidity was calculated.

The enzyme activity unit was defined as the reciprocal of the dilution at 37°C, after 30 min, which could have reduced the turbidity of the bacterium by 50%. The Ab32 was removed the bacterial outer membrane by 10% trichloromethane. The OD600 of Ab32 which suspended in PBS was 1. The enzyme activity unit was determined by turbidimetric regression. The bacterial suspension was added to the 96-well plate, 100 μL per well. A volume of 100 μL of a double dilution of endolysin Ply6A3, with an initial concentration of 3.9 mg/mL, was added to each well. Absorbance was measured at 600 nm before and after incubation. Based on the two readings, the percentage drop in bacterial turbidity was calculated (Oliveira et al., 2016).

### Lytic Spectrum of Endolysin Ply6A3 Against Clinical ESKAPE Isolates

The acronym ESKAPE stands for bacterial pathogens and include *E. faecium*, *S. aureus*, *K. pneumoniae*, *A. baumannii*, *P. aeruginosa*, and *Enterobacter*. The lytic spectrum of endolysin Ply6A3 against the 440 clinical isolates were determined by the plate method (Zhang et al., 2016). The clinical strains were suspended in 300 μL PBS (pH 7.4), to yield a final concentration of 1 × 10^9^ cfu/mL. The cells were plated with 4 mL agar (Lood et al., 2015). After solidification, the gel was perforate with a sterilizing puncher. A volume of 20 μL endolysin Ply6A3 (1 mg/mL) was added into a hole. The phage PD-6A3 (1 × 10^8^ PFU/mL), cocktail (1 × 10^8^ PFU/mL) which contain the 14 phages and the PBS were added into three other holes as comparison. The mixtures were cultured at 37°C overnight.

### Phage Therapy in the Mouse Sepsis Model

We investigated the ability of the phage PD6A3, phage cocktail (contain all the 14 phages) and endolysin Ply6A3, after removed endotoxin by the EndotoxinOUT kit (G-bioscience, United States), to work systemically to rescue sepsis in mice. Briefly, 80 6∼8 weeks-old female BALB/c mice (Shanghai Laboratory Animal Company, China) were randomly assigned to eight groups (*n* = 10 per group) (Jun et al., 2014). The first three groups were inoculated intraperitoneally with 1 mL 1 × 10^9^ CFU/mL of AB32 (minimum lethal dose, Supplementary Figure 2) and intraperitoneal administration with either 1 mL of endolysin Ply6A3 at 2 mg/mL (10 mice), 1 mL of phage PD6A3 (10^9^ PFU/mL), or 1 mL of PBS (10 mice), 1 h later. The remaining five groups were only inoculated intraperitoneally with 1 mL PBS, 1 mL of endolysin Ply6A3 at 2 mg/mL, 1 mL of phage PD6A3 (10^9^ PFU/mL), 1 mL of phage Cocktail (10^9^ PFU/mL) or 1ml of the mixture [0.5 mL of endolysin Ply6A3 at 2 mg/mL + 0.5 mL of phage PD6A3 (10^9^ PFU/mL)] respectively. The animals were monitored for 7 days and the survival rate of each group was calculated. The white blood cell (WBC) count, levels of IL-10 (interleukin-10) and PCT (procalcitonin) were evaluated to assess the immune status (Henein et al., 2016). At 2 days post-infection, three mice were selected from each group and subjected to 0.1 mL pentobarbital sodium (10 g/mL) anesthesia by intraperitoneal injection. Blood was collected from each anesthetized mouse then was sent to the clinical laboratory for examination of the WBC. The serum for measurement of IL-10 and PCT (Entenza et al., 2005) were used DuoSet enzyme-linked immunosorbent assay kits (R&D Systems). All the mice were sacrificed by cervical dislocation after experiment.

### Statistical Analysis

Statistical calculations for mice survival rate were performed by Kaplan–Meier survival analysis with log-rank test (GraphPad Prism 7.0 software), and the comparison of cytokine levels between groups were analyzed statistically by Student’s *t*-test (SPSS Statistics 21.0 software). *P*-values < 0.05 were considered statistically significant.

### Ethics Statement

This study was carried out in accordance with the recommendations of “Animal experiment guidelines, the Animal Care Committee of Fudan University.” The protocol was approved by the ‘the Animal Care Committee of Fudan University.’

## Results

### Isolation and Lytic Spectrum of *A. baumannii* Phages

About 40 different virulent phages were isolated, and 14 of those were additionally tested host spectra. The 14 phages were designated as PD-Ab1, PD-Ab8, PD-Ab9, PD-Ab11, PD-Ab 15, PD-Ab16, PD-Ab17, PD-Ab18, PD-6A1, PD-6A2, PD-6A3, PD-6A4, PD-7A1, and PD-7Ab3 (Supplementary Table 2). The results showed that all the phages have completely different lytic spectrum. Compared with the other 13 phages, phage PD-6A3 had a broader host range 32.4% (179/552) of the isolates. The cocktail which contains all the 14 phages was capable of lyzing 54.0% (298/552) of the isolates. Thus, we chose phage PD-6A3 for further study.

**Figure 1:**
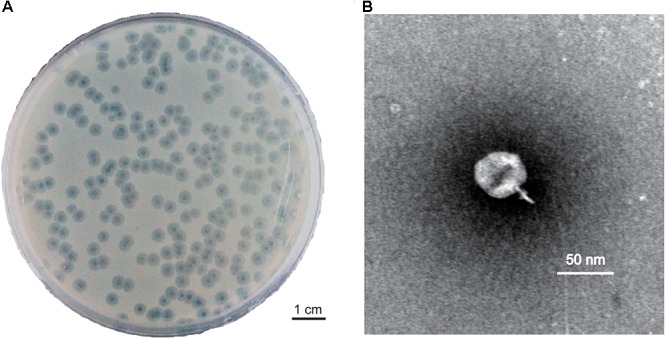
Plaque and TEM morphology of phages. **(A)** Plaque morphologies of phage PD-6A3. Scale bar, 1 cm. **(B)** TEM morphology of phage PD-6A3. Scale bar, 50 nm. PD-6A3, a novel *Acinetobacter baumannii* Phage. TEM, transmission electron micrograph.

Phage PD-6A3 formed clear, round, significant halos, 2–3 mm in diameter (Figure 1A). TEM showed that phage PD-6A3 possessed an isometric head (50 ± 10 nm in diameter) and a very short tail (9 ± 2 nm in length), which was nearly invisible (Figure 1B). The phage was assigned to the order Caudovirales and family Podoviridae.

### Biological Characterization and Lytic Effect of Phage PD-6A3

**Figure 2:**
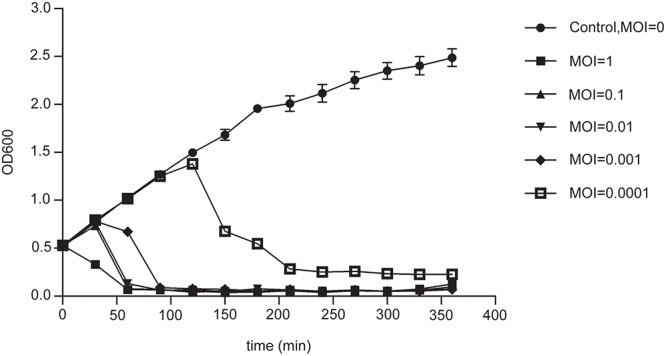
The infection assay of phage PD-6A3 against *A. baumannii in vitro*. AB32 was infected by phage PD6A3 at MOI of 0, 1, 0.1, 0.01, 0.001 or 0.0001 and cultured for 7 h. The control experiments were performed using equal volume of SM buffer. This experiment was repeated three times, and the data were shown in the mean ± SD. PD-6A3, a novel *Acinetobacter baumannii* Phage.

The stability of phage PD-6A3 was investigated at different temperatures and pH conditions. No significant loss in phage activity was observed after heating to temperatures between 4 and 50°C. In addition, it remained relatively stable after incubation at pH levels ranging from 5 to 10 (Supplementary Figures 1A,B). The adsorption experiment showed that more than 90% of the phage particles were adsorbed within 5 min incubation (Supplementary Figure 1C). One-step growth experiments showed that the latent and eclipse periods were both 20 min and the burst size was 129 PFU per infected cell (Supplementary Figure 1D). The infection assay of phage PD-6A3 against AB32 was evaluated *in vitro*. The results showed that phage PD-6A3 could effectively reduce growth of the host bacteria, with OD values declining more quickly at MOI 1 than at MOI 0. 1, 0.01, or 0.001 (Figure 2).

### Sequencing and Bioinformatics Analysis of Phage PD-6A3 Genomic DNA

The phage PD-6A3 is a linear double-stranded DNA with a length of 41,563 bp and GC content of 39.48% (GenBank accession No. KT388102.1). A total of 48 putative genes were identified by GeneMarks, on the positive strand (Supplementary Table 3). The average length of a gene was 776 bp. Genome analysis also reviewed that this phage carried no virulence and antibiotic resistance genes which assure its safety to be applied for phage therapy in the future.

**Figure 3:**
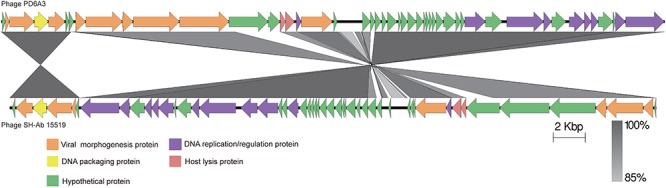
The comparison of the complete genome sequence of the Phage PD6A3 using Easy fig against the closet homolog phage SH-AB15519. The colored arrows indicate ORFs according to their predicted function. The homologous regions between phages are indicated by gray shading.

The proteins encoded by phage PD-6A3 could be divided into five categories morphogenesis, DNA packing protein, host lysis, DNA replication/regulation, and hypothetical proteins (Figure 3). According to the NCBI database, phage PD-6A3 has similarities with *Acinetobacter* phage SH-Ab15519 (GenBank accession number: KY082667.1; 99% identity, 93% coverage). The relatedness of these two phages was also determined using the Easy fig software show that phage PD-6A3 shares only 22 completely identical proteins with *Acinetobacter* phage SH-Ab15519, which is relatively limited (Figure 3). These all showed that Phage PD6A3 is a novel phiKMV-like phage.

**Figure 4:**
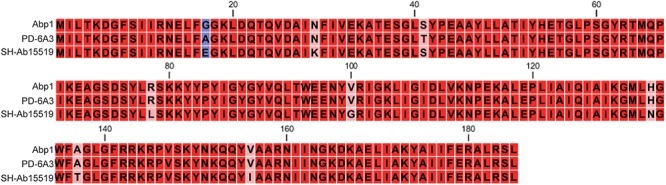
Amino acid sequence alignment of the endolysin of Phage PD6A3. The sequences used for alignment analysis were the endolysin gene of Phage 6A3, Phage SH-Ab15519 and Phage Abp1. Eight mutation residues are indicated by different color.

The endolysin gene of the phage PD6A3 is 558 bp and contains 185 amino acid residues (22.4 kDa protein), which belongs to glycoside hydrolase family 19. Conserved domain analysis has revealed the presence of alysozyme-like (*N*-acetyl-*b*-D-muramidase) catalytic domain between residues 75 and 128. A ClustalX2 alignment of endolysin Ply6A3 with two phage endolysins showed similarity in the domain region, however, 8 amino acid mutations were found (Figure 4).

### Cloning, Expression, Purification, and Assembly of Endolysin Ply6A3

**Figure 5:**
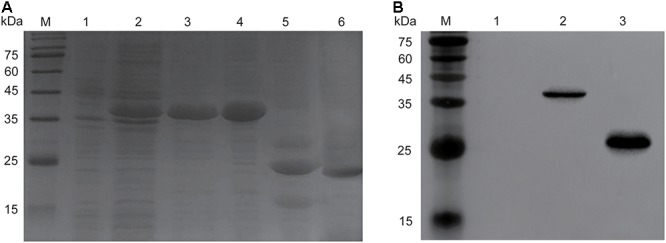
Over expression and purification of endolysin Ply6A3. **(A)** Lane M, molecular weight marker; Lane 1, un-induced bacterial lysate; Lane 2, IPTG-induced bacterial lysate; Lane 3, purified His-SUMO-Ply6A3 protein; Lane 4, dialyzed His-SUMO-Ply6A3 protein; Lane 5, Ply6A3 protein after cleavage of the His-SUMO tag; Lane 6, purified endolysin Ply6A3 protein after cleavage and dialyze of the His-SUMO tag. **(B)** Lane M, molecular weight marker; Lane 1 un-induced bacterial lysate; Lane 2-4, IPTG-induced bacterial lysate; Lane 5, the His-SUMO tag cleaved from endolysin Ply6A3. Ply6A3, the endolysin encoded by a novel *Acinetobacter baumannii* Phage *PD6A3* gene. IPTG, Isopropyl β-D-Thiogalactoside.

The molecular weight of recombinant endolysin Ply6A3 is approximately 40 kDa (Figure 5A). A specific band (His-SUMO tag) of approximately 23 kDa was identified using specific His-tag antibodies (Figure 5B). The concentration of purified endolysin reached about 3.9 mg/mL.

### Detection of Peptidoglycan Degradation by Endolysin PlyAB3 Using the Diffusion Method

**Figure 6:**
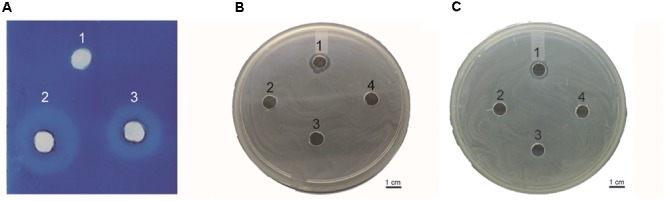
**(A)** Detection of peptidoglycan degradation by endolysin Ply6A3 by diffusion method. Hole 1: 20 μl PBS, Hole 2: 20 μl Lysozyme (2 mg/ml), Hole 3: 20 μl endolysin (2 mg/ml). **(B)** Lytic activity of endolysin against clinical AB32 isolates. **(C)** Lytic activity of endolysin Ply6A3 against clinical MRSA6 isolates. Hole 1: 20 μl endolysin (2 mg/ml), Hole 2: 20 μl Phage PD6A3 (10^8^cuf/ml), Hole 3: 20 μl Phage Cocktail (10^8^cuf/ml) Hole 4: 20 μl PBS. Scale bar, 1 cm. CFU, colony-forming units. PD6A3, a novel *Acinetobacter baumannii* Phage. Ply6A3, the endolysin encoded by a novel *Acinetobacter baumannii* Phage *PD6A3* gene. AB32, the No. 32 strains of Multidrug Resistance *Acinetobacter baumannii.* MRSA6, the No. 6 strains of methicillin-resistant *Staphylococcus aureus*.

This assay was performed to determine whether the endolysin Ply6A3 indeed with lytic activities toward cell walls (Lai et al., 2013). According to the principle of methylene blue staining, peptidoglycan stains blue (Figure 6A). Endolysin Ply6A3 (2 mg/mL) and lysozyme (2 mg/mL) can degrade peptidoglycan to form a transparent ring; whereas the negative control, PBS, showed no degradation and formed no transparent rings.

### Cytotoxicity of Phage PD6A3 and Endolysin Ply6A3 to HEK293T and THP-1 Cells

**Figure 7:**
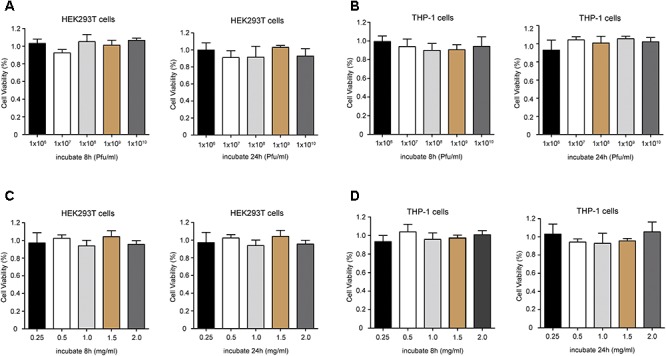
Cell viability of HEK293T and THP-1 cells after incubation with different concentrations of Phage PD6A3 **(A,B)** and endolysin Ply6A3 **(C,D)**. Cells were incubated with different concentrations of phage PD-6A3 (10^6^–10^10^ Pfu/mL) and endolysin Ply6A3 (0.25–2 mg/mL) for 8 h then followed by fresh complete media for 16 h. Or only incubated with different concentrations of phage PD-6A3 (10^6^–10^10^ Pfu/mL) and endolysin Ply6A3 (0.25–2 mg/mL) for 24 h. Cell viability was measured using the CCK-8. Data are mean ± SD (*n* = 6). All the result shows that cell lines remained highly viable after incubation with endolysin Ply6A3. Ply6A3, the endolysin encoded by a novel *Acinetobacter baumannii* Phage *PD6A3* gene. HEK293T cells, human embryonic kidney cells 293. THP-1 cells, a human monocytic cell line derived from an acute monocytic leukemia patient. CCK-8, Cell Counting Kit-8.

The potential cytotoxicity of phage PD6A3 were evaluated using the HEK293T and THP-1cells by the CCK-8 assay. The results showed that the percentage of viable HEK293T cells were around 100%, treated with Phage PD6A3 in 8 h and 24 h. We changed the cells to THP-1, and the result is the same (Figures 7A,B). And no significant cytotoxicity was observed in either HEK293T or THP-1 cells, treated with endolysin Ply6A3 (Figures 7C,D) (Shen et al., 2018; Yin et al., 2018).

### Biological Characterization and Lytic Effect of Endolysin Ply6A3

**Figure 8:**
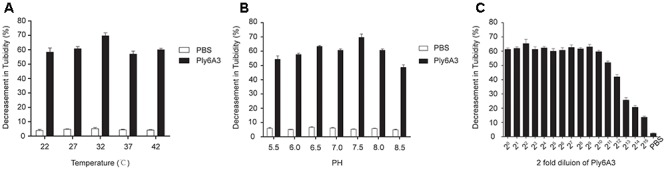
**(A)** The thermal stability of endolysin Ply6A3. Endolysin was incubated at various temperatures for 30 min. **(B)** The pH stability of endolysin Ply6A3. Endolysin was incubated at the indicated pH conditions for 30 min. **(C)** Activity unit of endolysin Ply6A3. The initial concentration was 3.9 mg/mL, then double dilution. Data were obtained from three independent experiments and are shown as mean ± SD. Ply6A3, the endolysin encoded by a novel *Acinetobacter baumannii* Phage *PD6A3* gene. pH, hydrogen ion concentration. AB32, the No. 32 strains of Multidrug Resistance *Acinetobacter baumannii.*

The temperature and pH stability test revealed that endolysin Ply6A3 remained high activity at temperature between 22 and 42°C and at pH range from 5.5 to 8.5; however, its activity was significantly reduced at pH levels above 7.5 (Figures 8A,B). The broad range of thermal and pH stability facilitates the application of endolysin in phage therapy.

The lytic effect shown that, when the original concentration of endolysin Ply6A3 was 3.9 mg/mL, its dilution ratio was 1:2^11^, and the turbidity of AB32 could have been reduced by nearly 50% (Figure 8C). The results showed that the activity unit of endolysin Ply6A3 was 2048 unit/mL or 525 unit/mg. The amount of endolysin protein contained in each active unit was 1.9 μg. After fitting to a function curve, the IC50 of endolysin was calculated to be 1.6 μg.

### Lytic Spectrum of Endolysin Ply6A3

Lytic spectrum was determined by spot test, the results show that 141 of the 200 MDRAB strains could have been lysed by endolysin Ply6A3 (Table 2). Moreover, 21 of the 40 PDR-AB strains could have been lysed. In addition, the protein also showed lytic activity for other septic bacterial strains as follows: *E. faecium*, *S. aureus*, *K. pneumonia*, and *E. coli* strains. The lytic spectrum of endolysin Ply6A3 is broader than that of phage PD6A3 and phage Cocktail which contain 14 phages (Table 3). None of the 40 *P. aeruginosa* strains could have been lysed. Only one hole (added endolysin Ply6A3) formed a bright, clear inhibition zone (Figures 6B,C).

**Table 2:** Lysis spectrum of endolysin Ply-6A3.

Bacteria	Positive	Total	Positive rate (%)
*Enterococcus faecium*	5	40	12.5
*Staphylococcus aureus*	9	40	22.5
*Klebsiella pneumonia*	3	40	7.5
*Pseudomonas aeruginosa*	0	40	0
*Escherichia coli*	8	40	20.0
MDR-*A. baumannii*	141	200	70.5
PDR-*A. baumannii*	21	40	52.5

*MDR-A. baumannii*, multi-drug resistant *Acinetobacter baumannii*. PDR-*A. baumannii*, pan-drug resistant *Acinetobacter baumannii*.

**Table 3:** Lysis spectrum of endolysin Ply-6A3 and Phage PD6A3 toward 200 MDR-AB strains.

	Positive	Total	Positive rate (%)
Ply6A3	141	200	70.5
Phage PD6A3	58	200	29.0
Cocktail	90	200	45.0

*PD6A3, a novel *Acinetobacter baumannii* Phage. Ply6A3, the endolysin encoded by a novel *Acinetobacter baumannii* Phage *PD6A3* gene. Cocktail, the cocktail which contain all the 14 phages mentioned in this article*.

### Phage Therapy in the Mouse Sepsis Model

**Figure 9:**
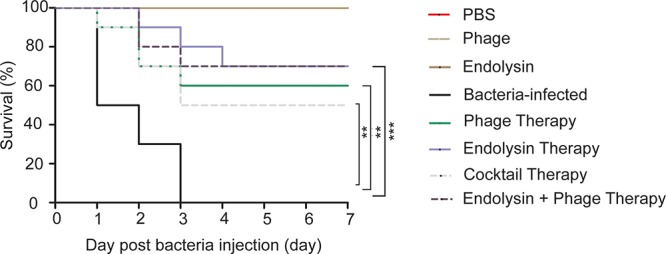
Therapeutic efficacy of Phage PD6A3 and endolysin Ply6A3 against mice *A. baumannii* sepsis infections. Survival of mice that were sepsis infected with AB32 and then treated with endolysin Ply6A3, phage PD6A3; or only intraperitoneal injected with PBS, Phage, endolysin. Using log-rank (Mantel-Cox) test. Each group harbors 10 mice. ^∗∗^*P* < 0.01, ^∗∗∗^*P* < 0.001 compared with the Bacteria group. PD6A3, a novel *Acinetobacter baumannii* Phage. Ply6A3, the endolysin encoded by a novel *Acinetobacter baumannii* Phage *PD6A3* gene. AB32, the No. 32 strains of Multidrug Resistance *Acinetobacter baumannii*.

To ascertain the effect of phage and endolysin against the sepsis mouse model, mouse was developed by intraperitoneal administration of *A. baumannii.* The survival rate of endolysin therapy group, endolysin + phage therapy group, phage therapy group and phage cocktail therapy group were 70, 70, 60, and 50%, respectively, higher than the bacterial group (Figure 9). Mice in the endolysin therapy group had milder clinical signs (slow reactions, lethargy, and activity reduction) than the mice in other three therapy groups. Furthermore, all mice in the negative control group (injected with PBS, phage or endolysin respectively) remained alive after 7 days.

**Figure 10:**
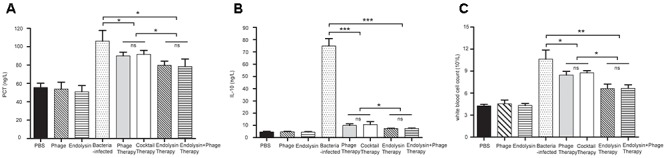
Levels of WBC and cytokines in the blood of the different groups of mice. The blood of the mice in the groups of PBS, phage, endolysin, bacteria-infected, Phage therapy, Phage cocktail therapy, Endolysin therapy and Endolysin + phage therapy were collected at 2 days. **(A)** PCT and **(B)** IL-10 concentrations of serum were measured by ELISA. **(C)** Blood was sent to the clinical laboratory to examine WBC. The data were shown in the mean ± SD (*n* = 3). ^∗^*P* < 0.05, ^∗∗^*P* < 0.01, and ^∗∗∗^*P* < 0.001. WBC, white blood cell count. IL-10, interleukin-10. PCT, procalcitonin.

On the second day of the experiment, WBC counts in all the 4 therapy groups were significantly lower than those in the sepsis control group (*P* < 0.05). Furthermore, WBC counts in the endolysin therapy group and endolysin + phage therapy group were significantly lower than those in the phage therapy group and phage cocktail group (*P* < 0.05). The levels of IL-10 and PCT were significantly elevated in sepsis group, while all the 4 therapy groups effectively reduced the level. The expression of WBC, IL-10 and PCT remained at basal levels in the mice from control, phage group and endolysin group (Figure 10).

## Discussion

*Acinetobacter baumannii* is an important nosocomial pathogen worldwide. This bacterium can be easily spread throughout healthcare facilities (Kusradze et al., 2016). The treatment of *A. baumannii* infection becomes increasingly difficult because of its resistance to many antimicrobial agents. Moreover, phages and their endolysins have drawn the attention of researchers to explore their potential as antimicrobial agents, because of the low probability of developing resistance, effectiveness against bacteria both *in vitro* and *vivo*, and ease of access (Oliveira et al., 2016).

The use of phages in animals has been reported in various applications, such as in the treatment of lung infections and skin wounds, and the results of such therapy have been significant (Lood et al., 2015; Shivaswamy et al., 2015; Henein et al., 2016). Hua and colleagues used an *A. baumannii* phage to rescue carbapenem-resistant *A. baumannii* (CRAB) lung infections (Hua et al., 2018). The use of endolysin treatments in animals is less common than that of phages. Zhang et al. (2016) proved the safety and efficacy of *Streptococcus suis* phage endolysin against *Streptococcus suis* infection in mice (Zhang et al., 2016). These experiments demonstrate that the phage and its endolysin could provide a promising future in the quest for treatments against bacterial infections. However, these studies have some limitations as follows: only a few resistant strains were used to test the lytic spectrum; all strains were collected from one hospital; the lack of a specific, effective concentration of the endolysin; and the lack of quantitative analysis with reliance on qualitative analysis. These limitations all render the reported lytic rates less convincing (Huang et al., 2014; Defraine et al., 2016; Larpin et al., 2018).

We isolated 40 *A. baumannii* phages, among which phage PD-6A3 had a relatively broad host range of 32.4% (179/552) among clinical MDR *A. baumannii* isolates, and was selected for further study. Phage PD-6A3 showed outstanding characteristics compared to the other phages such as remained stable over a wide range of temperatures and pH conditions. Genome analysis also reviewed that this phage carried no virulence and antibiotic resistance genes. Considering these advantages, phage PD-6A3 proves to be promising for potential applications in *A. baumannii* infections.

We over-expressed and purified the endolysin. The final concentration of endolysin Ply6A3 was 3.9 mg/mL. Previous studies have rarely included quantitative descriptions. The endolysin remained stable over a wide range of temperatures and pH conditions. Our results demonstrate that as a murein hydrolase, endolysin Ply6A3 was able to degrade 141/200 clinical MDRAB isolates and 21/40 PDR-AB isolates. In addition, it was also able to degrade Enterococci, MRSA, *K. pneumonia*, MDR- *P. aeruginosa*, and ESBL-*E. coli*, but Phage PD6A3 could not lysis any of them. Interestingly, we also over-expressed the endolysin of Phage Ab9 (GenBank accession number KY388103.), but it only could degrade 18/200 clinical MDRAB isolates which was very narrow compared to endolysin Ply6A3. In our future study, we will analysis this phenomenon in depth. Compared with previous articles, our research group tested the greatest number of bacterial strains, including *A. baumannii* and other common pathogens (Lai et al., 2013). Interestingly, when endolysin Ply6A3, phage PD6A3 and phage cocktail acted on 200 MDRAB strains, the lytic spectrum of the endolysin was much broader than the other groups. This might be attributed to different features of the outer membrane structure, like a different composition in the lipopolysaccharides, which may prevent or facilitate the access to exogenous lysins (Huang et al., 2014). We are further studying this phenomenon.

We extracted the cell wall of *A. baumannii* (Endersen et al., 2015). The results demonstrated that the peptidoglycan can be degraded by endolysin Ply6A3 and lysozyme. However, only endolysin could lyse *A. baumannii* strains, whereas lysozyme could not. These findings indicate that endolysin Ply6A3 is exceptional in its ability to directly destroy the cell wall of Gram-positive bacteria. These findings are also consistent with previous reports of the ability of endolysins from Gram-negative phages to lyse bacterial cells through enzymatic activity.

Our research also indicated the safety of phage PD6A3 and endolysin Ply6A3 applied intraperitoneal injection for mice. No obvious side effect was observed after injection during the observation period. And no statistical significance was observed when compared cytokine levels in serum, WBC in blood of the phage group and endolysin group to the PBS group, which provided a further safety evidence for the phage therapy and endolysin therapy by intraperitoneal injection. If in combination, phage PD-6A3 and endolysin Ply6A3 had more activity than the cocktail of 14 phages.

Compared to the sepsis group, all the therapy groups showed significantly higher survival rates and with evident reductions in the WBC count, serum PCT, and serum IL-10. However, the difference in survival between the 4 therapy groups were not statistically significant (*P* > 0.05). This might because the relatively small sample size could have affected the accuracy of statistical analysis. On the second day of the experiment showed that the WBC count, serum IL-10 and PCT of the endolysin therapy group and endolysin + phage therapy group were lower than that of the phage therapy group and cocktail therapy group (*P* < 0.05), which implies that endolysins work faster than phages. However, these are just experimental proofs that not very close to clinical situation (such short an interval time in clinical practice is unlikely). And the outcome of endolysin Ply6A3, as a class of enzymes, after entering the body is not clear. These conclusions need to be verified through further studies especially supported by clinical trials (Takemura-Uchiyama et al., 2014; Regeimbal et al., 2016).

Taken together, phage PD-6A3 is a novel phiKMV-like phage with strong lytic activity. Its endolysin Ply6A3 is an effective antibiotic against Gram-negative and Gram-positive bacteria. In addition, we evaluated the safety, effectiveness, and routes of administration for phage PD6A3 and its endolysin Ply6A3, in the treatment of *A. baumannii* infections, both *in vivo* and *in vitro*. Both agents have potentially therapeutic against infections caused by MDRAB strains in the future.

## Author Contributions

YS and DC designed and supervised the study. MW, YL, DM, HG, and ZZ performed the experiments, analyzed the data, and drafted the manuscript. KH and YZ performed the experiments and analyzed the data. YX provided advice and suggestions.

## Conflict of Interest Statement

The authors declare that the research was conducted in the absence of any commercial or financial relationships that could be construed as a potential conflict of interest.

## Footnotes

1 http://blast.ncbi.nlm.nih.gov/Blast.cgi

2 https://web.expasy.org/compute_pi/

## References

[B1] Adams M. H. (1959). *Bacteriophages.* New York, NY: Interscience Publishers.

[B2] Billiau A. (2016). At the centennial of the Bacteriophage: reviving the overlooked contribution of a forgotten pioneer. Richard Bruynoghe (1881–1957). *J. Hist. Biol.* 49 559–580. 10.1007/s10739-015-9429-0 26515105

[B3] Blázquez B. Fresco-Taboada A. Iglesias-Bexiga M. Menéndez M. García P. (2016). PL3 amidase, a tailor-made lysin constructed by domain shuffling with potent killing activity against Pneumococci and related species. *Front. Microbiol.* 7:1156. 10.3389/fmicb.2016.01156 27516758 PMC4963390

[B4] Capra M. L. Quiberoni A. Reinheimer J. A. (2004). Thermal and chemical resistance of *Lactobacillus casei* and *Lactobacillus paracasei* bacteriophages. *Lett. Appl. Microbiol.* 38 499–504. 10.1111/j.1472-765X.2004.01525.x 15130146

[B5] CLSI (2017). “Performance standards for antimicrobial susceptibility testing,” in *Proceedings of the 27th Informational Supplement M100-S20* (Wayne, PA: Clinical and Laboratory Standards Institute).

[B6] Defraine V. Schuermans J. Grymonprez B. Govers S. K. Aertsen A. Fauvart M. (2016). Efficacy of artilysin Art-175 against resistant and persistent *Acinetobacter baumannii*. *Antimicrob. Agents Chemother.* 60 3480–3488. 10.1128/AAC.00285-16 27021321 PMC4879360

[B7] Diez-Martinez R. De Paz H. D. Garcia-Fernandez E. Bustamante N. Euler C. W. Fischetti V. A. (2015). A novel chimeric phage lysin with high in vitro and in vivo bactericidal activity against *Streptococcus pneumoniae*. *J. Antimicrob. Chemother.* 70 1763–1773. 10.1093/jac/dkv038 25733585

[B8] Doi Y. Murray G. Peleg A. (2015). *Acinetobacter baumannii*: evolution of antimicrobial resistance—treatment options. *Semin. Res. Crit. Care* 36 085–098. 10.1055/s-0034-1398388 25643273 PMC4465586

[B9] El-Shibiny A. El-Sahhar S. (2017). Bacteriophages: the possible solution to treat infections caused by pathogenic bacteria. *Can. J. Microbiol.* 63 865–879. 10.1139/cjm-2017-0030 28863269

[B10] Endersen L. Coffey A. Ross R. P. McAuliffe O. Hill C. O’Mahony J. (2015). Characterisation of the antibacterial properties of a bacterial derived peptidoglycan hydrolase (LysCs4), active against *C. sakazakii* and other Gram-negative food-related pathogens. *Int. J. Food Microbiol.* 215 79–85. 10.1016/j.ijfoodmicro.2015.08.007 26342306

[B11] Entenza J. M. Loeffler J. M. Grandgirard D. Fischetti V. A. Moreillon P. (2005). Therapeutic effects of bacteriophage Cpl-1 lysin against *Streptococcus pneumoniae* endocarditis in rats. *Antimicrob. Agents Chemother.* 49 4789–4792. 10.1128/AAC.49.11.4789-4792.2005 16251333 PMC1280127

[B12] Guo H. Zhu J. Tan Y. Li C. Chen Z. Sun S. (2016). Self-assembly of virus-like particles of rabbit hemorrhagic disease virus capsid protein expressed in *Escherichia coli* and their immunogenicity in rabbits. *Antiviral Res.* 131 85–91. 10.1016/j.antiviral.2016.04.011 27118636

[B13] Henein A. E. Hanlon G. W. Cooper C. J. Denyer S. P. Maillard J. (2016). A partially purified *Acinetobacter baumannii* phage preparation exhibits no cytotoxicity in 3T3 mouse fibroblast cells. *Front. Microbiol.* 7:1198. 10.3389/fmicb.2016.01198 27536286 PMC4971803

[B14] Hu F. P. Guo Y. Zhu D. M. Wang F. Jiang X. F. Xu Y. C. (2016). Resistance trends among clinical isolates in China reported from CHINET surveillance of bacterial resistance, 2005–2014. *Clin. Microbiol. Infect.* 22 S9–S14. 10.1016/j.cmi.2016.01.001 27000156

[B15] Hua Y. Luo T. Yang Y. Dong D. Wang R. Wang Y. (2018). Phage therapy as a promising new treatment for lung infection caused by carbapenem-resistant *Acinetobacter baumannii* in mice. *Front. Microbiol.* 8:2659. 10.3389/fmicb.2017.02659 29375524 PMC5767256

[B16] Huang G. Le S. Peng Y. Zhao Y. Yin S. Zhang L. (2013). Characterization and genome sequencing of phage Abp1, a new phiKMV-like virus infecting multidrug-resistant *Acinetobacter baumannii*. *Curr. Microbiol.* 66 535–543. 10.1007/s00284-013-0308-7 23328903

[B17] Huang G. Shen X. Gong Y. Dong Z. Zhao X. Shen W. (2014). Antibacterial properties of *Acinetobacter baumannii* phage Abp1 endolysin (PlyAB1). *BMC Infect. Dis.* 14:681. 10.1186/s12879-014-0681-2 25495514 PMC4274762

[B18] Jin J. Li Z. J. Wang S. W. Wang S. M. Huang D. H. Li Y. H. (2012). Isolation and characterization of ZZ1, a novel lytic phage that infects *Acinetobacter baumannii* clinical isolates. *BMC Microbiol.* 12:156. 10.1186/1471-2180-12-156 22838726 PMC3438129

[B19] Jun S. Y. Jung G. M. Yoon S. J. Choi Y. Koh W. S. Moon K. S. (2014). Preclinical safety evaluation of intravenously administered SAL200 containing the recombinant phage endolysin SAL-1 as a pharmaceutical ingredient. *Antimicrob. Agents Chemother.* 58 2084–2088. 10.1128/AAC.02232-13 24449776 PMC4023757

[B20] Khakhum N. Yordpratum U. Boonmee A. Tattawasart U. Rodrigues J. L. M. Sermswan R. W. (2016). Cloning, expression, and characterization of a peptidoglycan hydrolase from the *Burkholderia pseudomallei* phage ST79. *AMB Express* 6:77. 10.1186/s13568-016-0251-7 27637947 PMC5025407

[B21] Kitti T. Thummeepak R. Thanwisai A. Boonyodying K. Kunthalert D. Ritvirool P. (2014). Characterization and detection of endolysin gene from three *Acinetobacter baumannii* bacteriophages isolated from sewage water. *Indian J. Microbiol.* 54 383–388. 10.1007/s12088-014-0472-x 25320435 PMC4186927

[B22] Kong M. Ryu S. (2015). Bacteriophage PBC1 and its endolysin as an antimicrobial agent against *Bacillus cereus*. *Appl. Environ. Microbiol.* 81 2274–2283. 10.1128/AEM.03485-14 25595773 PMC4357929

[B23] Kusradze I. Karumidze N. Rigvava S. Dvalidze T. Katsitadze M. Amiranashvili I. (2016). Characterization and testing the efficiency of *Acinetobacter baumannii* phage vB-GEC_Ab-M-G7 as an antibacterial agent. *Front. Microbiol.* 7:1590. 10.3389/fmicb.2016.01590 27757110 PMC5047890

[B24] Lai M. Lin N. Hu A. Soo P. Chen L. Chen L. (2011). Antibacterial activity of *Acinetobacter baumannii* phage ϕAB2 endolysin (LysAB2) against both Gram-positive and Gram-negative bacteria. *Appl. Microbiol. Biotechnol.* 90 529–539. 10.1007/s00253-011-3104-y 21264466

[B25] Lai M. Soo P. Lin N. Hu A. Chen Y. Chen L. (2013). Identification and characterisation of the putative phage-related endolysins through full genome sequence analysis in *Acinetobacter baumannii* ATCC 17978. *Int. J. Antimicrob. Agents* 42 141–148. 10.1016/j.ijantimicag.2013.04.022 23742833

[B26] Larpin Y. Oechslin F. Moreillon P. Resch G. Entenza J. M. Mancini S. (2018). In vitro characterization of PlyE146, a novel phage lysin that targets Gram-negative bacteria. *PLoS One* 13:e0192507. 10.1371/journal.pone.0192507 29408864 PMC5800649

[B27] Lim J. Shin H. Heu S. Ryu S. (2014). Exogenous lytic activity of SPN9CC endolysin against gram-negative bacteria. *J. Microbiol. Biotechnol.* 24 803–811. 10.4014/jmb.1403.03035 24690638

[B28] Lin M. (2014). Antimicrobial resistance in*Acinetobacter baumannii* : from bench to bedside. *World J. Clin. Cases* 2:787. 10.12998/wjcc.v2.i12.787 25516853 PMC4266826

[B29] Lood R. Winer B. Y. Pelzek A. J. Diez-Martinez R. Thandar M. Euler C. W. (2015). Novel phage lysin capable of killing the multidrug-resistant gram-negative bacterium *Acinetobacter baumannii* in a mouse Bacteremia model. *Antimicrob. Agents Chemother.* 59 1983–1991. 10.1128/AAC.04641-14 25605353 PMC4356752

[B30] Melo L. D. R. Brandao A. Akturk E. Santos S. B. Azeredo J. (2018). Characterization of a new *Staphylococcus aureus* kayvirus harboring a lysin active against biofilms. *Viruses* 10:182. 10.3390/v10040182 29642449 PMC5923476

[B31] Oliveira H. Vilas Boas D. Mesnage S. Kluskens L. D. Lavigne R. Sillankorva S. (2016). Structural and enzymatic characterization of ABgp46, a novel phage endolysin with broad anti-gram-negative bacterial activity. *Front. Microbiol.* 7:208. 10.3389/fmicb.2016.00208 26955368 PMC4768612

[B32] Pajunen M. Kiljunen S. Skurnik M. (2000). Bacteriophage phiYeO3-12, specific for *Yersinia enterocolitica* serotype O:3, is related to coliphages T3 and T7. *J. Bacteriol.* 182 5114–5120. 10.1128/JB.182.18.5114-5120.2000 10960095 PMC94659

[B33] Regeimbal J. M. Jacobs A. C. Corey B. W. Henry M. S. Thompson M. G. Pavlicek R. L. (2016). Personalized therapeutic cocktail of wild environmental phages rescues mice from *Acinetobacter baumannii* wound infections. *Antimicrob. Agents Chemother.* 60 5806–5816. 10.1128/AAC.02877-15 27431214 PMC5038255

[B34] Shen Y. Zhang J. Hao W. Wang T. Liu J. Xie Y. (2018). Copolymer micelles function as pH-responsive nanocarriers to enhance the cytotoxicity of a HER2 aptamer in HER2-positive breast cancer cells. *Int. J. Nanomed.* 13 537–553. 10.2147/IJN.S149942 29416334 PMC5790103

[B35] Shivaswamy V. C. Kalasuramath S. B. Sadanand C. K. Basavaraju A. K. Ginnavaram V. Bille S. (2015). Ability of bacteriophage in resolving wound infection caused by multidrug-resistant *Acinetobacter baumannii* in uncontrolled diabetic rats. *Microb. Drug Resist.* 21 171–177. 10.1089/mdr.2014.0120 25411824

[B36] Sun J. Shi Y. Le G. Ma X. (2005). Distinct immune response induced by peptidoglycan derived from *Lactobacillus* sp. *World J. Gastroenterool.* 11 6330–6337. 10.3748/wjg.v11.i40.6330 16419162 PMC4320337

[B37] Takemura-Uchiyama I. Uchiyama J. Osanai M. Morimoto N. Asagiri T. Ujihara T. (2014). Experimental phage therapy against lethal lung-derived septicemia caused by *Staphylococcus aureus* in mice. *Microbes Infect.* 16 512–517. 10.1016/j.micinf.2014.02.011 24631574

[B38] Yang H. Wang M. Yu J. Wei H. (2015). Antibacterial activity of a novel peptide-modified lysin against *Acinetobacter baumannii* and *Pseudomonas aeruginosa*. *Front. Microbiol.* 6:1471. 10.3389/fmicb.2015.01471 26733995 PMC4686776

[B39] Yele A. B. Thawal N. D. Sahu P. K. Chopade B. A. (2012). Novel lytic bacteriophage AB7-IBB1 of *Acinetobacter baumannii*: isolation, characterization and its effect on biofilm. *Arch. Virol.* 157 1441–1450. 10.1007/s00705-012-1320-0 22552486

[B40] Yin S. Huang G. Zhang Y. Jiang B. Yang Z. Dong Z. (2018). Phage abp1 rescues human cells and mice from infection by pan-drug resistant *Acinetobacter Baumannii*. *Cell. Physiol. Biochem.* 44 2337–2345. 10.1159/000486117 29258062

[B41] Zhang H. Zhang C. Wang H. Yan Y. X. Sun J. (2016). A novel prophage lysin Ply5218 with extended lytic activity and stability against *Streptococcus suis* infection. *FEMS Microbiol. Lett.* 363:fnw186. 10.1093/femsle/fnw186 27481700

