# Corrigendum: A Novel Phage PD-6A3, and Its Endolysin Ply6A3, With Extended Lytic Activity Against *Acinetobacter baumannii*

**DOI:** 10.3389/fmicb.2019.00196

**Published:** 2019-02-12

**Authors:** Minle Wu, Kongying Hu, Youhua Xie, Yili Liu, Di Mu, Huimin Guo, Zhifan Zhang, Yingcong Zhang, Dong Chang, Yi Shi

**Affiliations:** ^1^Department of Clinical Laboratory, Shanghai Pudong Hospital, Fudan University, Shanghai, China; ^2^Key Laboratory of Medical Molecular Virology, School of Basic Medical Sciences, Fudan University, Shanghai, China; ^3^Department of Clinical Laboratory, Shanghai Public Health Clinical Center, Shanghai, China; ^4^Department of Clinical Laboratory, Shanghai Fourth People's Hospital affiliated to Tongji University School of Medicine, Shanghai, China

**Keywords:** multidrug resistance *Acinetobacter baumannii*, endolysin, ESKAPE, sepsis, phage therapy, mice model

There is an error in the Funding statement. The correct name for the Funder is “Shanghai Municipal Commission of Health and Family Planning.”

Additionally, in the published article, there were errors in affiliations 1 and 4. Instead of “Department of Clinical Laboratory, Pudong Hospital Affiliated to Fudan University, Shanghai, China,” it should be “Department of Clinical Laboratory, Shanghai Pudong Hospital, Fudan University, Shanghai, China,” and in affiliation 4, instead of “Department of Clinical Laboratory, The Fourth People's Hospital of Shanghai, Shanghai, China,” it should be “Department of Clinical Laboratory, Shanghai Fourth People's Hospital affiliated to Tongji University School of Medicine, Shanghai, China.”

The authors apologize for these errors and state that this does not change the scientific conclusions of the article in any way. The original article has been updated.

