# Supplementary material for: A Novel Phage PD-6A3, and Its Endolysin Ply6A3, With Extended Lytic Activity Against Acinetobacter baumannii
Source: Front Microbiol. 2019 Jan 9;9:3302. doi: 10.3389/fmicb.2018.03302 (PMC6333635; doi:10.3389/fmicb.2018.03302)

**SUPPLEMENTAL MATERIAL**

**Table 1. The source of 552 clinical strains of Acinetobacter baumannii**

| Hospital | Number | Rate(%) |
| --- | --- | --- |
| Pudong Hospital Affiliated to Fudan University | 201 | 36.4 |
| Renji Hospital Affiliated to Shanghai Jiao Tong University | 212 | 38.4 |
| People's Hospital of Shanghai Pudong District of Shanghai | 62 | 11.2 |
| The Seventh People's Hospital of Shanghai University of TCM | 40 | 7.3 |
| Sixth People's Hospital Affiliated to Shanghai Jiao Tong University | 37 | 6.7 |

TCM: Traditional Chinese Medicine

**Table 2. Lytic Spectrum of 14 Single Phage and Phage Cocktail**

| Phage | Pudong Hospital | Renji Hospital | Sixth People's Hospital | Seventh People's Hospital | Pudong New Area People's Hospital | Total |
| --- | --- | --- | --- | --- | --- | --- |
| PD-AB1 | 16.9% | 10.4% | 8.1% | 17.5% | 24.2% | 14.7% |
|  | 34/201 | 22/212 | 3/37 | 7/40 | 15/62 | 81/552 |
| PD-AB8 | 14.4% | 9.9% | 2.7% | 5.0% | 12.9% | 11.1% |
|  | 29/201 | 21/212 | 1/37 | 2/40 | 8/62 | 61/552 |
| PD-AB9 | 25.9% | 13.7% | 24.3% | 40.0% | 37.1% | 23.4% |
|  | 52/201 | 29/212 | 9/37 | 16/40 | 23/62 | 129/552 |
| PD-AB11 | 3.5% | 0 | 0 | 0 | 0 | 1.3% |
|  | 7/201 | 0 | 0 | 0 | 0 | 7/552 |
| PD-AB15 | 15.4% | 11.8% | 0 | 0 | 4.8% | 10.7% |
|  | 31/201 | 25/212 | 0 | 0 | 3/62 | 59/552 |
| PD-AB16 | 16.4% | 12.3% | 0 | 0 | 4.8% | 11.2% |
|  | 33/201 | 26/201 | 0 | 0 | 3/62 | 62/552 |
| PD-AB17 | 26.9% | 21.7% | 2.7% | 5.0% | 19.4% | 20.8% |
|  | 54/201 | 46/212 | 1/37 | 2/40 | 12/62 | 115/552 |
| PD-AB18 | 14.9% | 11.3% | 10.8% | 5.0% | 14.5% | 12.5% |
|  | 30/201 | 24/212 | 4/37 | 2/40 | 9/62 | 69/552 |
| PD-6A1 | 24.9% | 23.6% | 8.1% | 55.0% | 37.1% | 26.8% |
|  | 50/201 | 50/212 | 3/37 | 22/40 | 23/62 | 148/552 |
| PD-6A2 | 13.4% | 11.8% | 2.7% | 5.0% | 14.5% | 11.6% |
|  | 27/201 | 25/212 | 1/37 | 2/40 | 9/62 | 64/552 |
| PD-6A3 | 33.8% | 23.1% | 43.2% | 55.0% | 38.7% | 32.4% |
|  | 68/201 | 49/212 | 16/37 | 22/40 | 24/62 | 179/552 |
| PD-6A4 | 25.4% | 25.5% | 8.1% | 57.5% | 38.7% | 28.1% |
|  | 51/201 | 54/212 | 3/37 | 23/40 | 24/62 | 155/552 |
| PD-7A1 | 28.4% | 31.6% | 8.1% | 57.5% | 38.7% | 31.5% |
|  | 57/201 | 67/212 | 3/37 | 23/40 | 24/62 | 174/552 |
| PD-7A3 | 28.4% | 31.6% | 8.1% | 57.5% | 38.7% | 31.5% |
|  | 57/202 | 67/212 | 3/37 | 23/40 | 24/62 | 174/552 |
| Cocktail | 60.7% | 46.2% | 56.8% | 60.0% | 53.2% | 54.0% |
|  | 122/201 | 98/212 | 21/37 | 24/40 | 33/62 | 298/552 |

**TABLE 3. Genomic annotation of PD-6A3**

| ORFs | Strand | LeftEnd | RightEnd | Codon | | Gene Length | Number of amino acids | Class | Description | Closet hit in NCBI | Coverage | % Amino acid identity | E-value | pI1 | MW2(Da) | Accession number |
| --- | --- | --- | --- | --- | --- | --- | --- | --- | --- | --- | --- | --- | --- | --- | --- | --- |
|  |  |  |  | Start | Stop |  |  |  |  |  |  |  |  |  |  |  |
| 1 | + | 73 | 270 | ATG | TGA | 198 | 65 | 1 | hypothetical protein | hypothetical protein vBAbaPPDAB9_19 [Acinetobacter phage vB_AbaP_PD-AB9] | 100 | 100 | 1E-40 | 6.18 | 7704.81 | YP_009189842.1 |
| 2 | + | 267 | 518 | ATG | TAG | 252 | 83 | 1 | Structural protein | structural protein [Acinetobacter phage vB_AbaP_PD-AB9] | 100 | 100 | 1E-53 | 6.21 | 9050.03 | YP_009189841.1 |
| 3 | + | 527 | 2083 | ATG | TAG | 1557 | 518 | 1 | Putative head-tail connector protein | putative head-tail connector protein [Acinetobacter phage vB_AbaP_PD-AB9] | 100 | 100 | 0 | 5.01 | 58828.39 | YP_009189840.1 |
| 4 | + | 2092 | 2952 | ATG | TAA | 861 | 286 | 1 | Putative scaffolding protein | putative scaffolding protein [Acinetobacter phage vB_AbaP_PD-AB9] | 100 | 100 | 0 | 5.51 | 30551.55 | YP_009189839.1 |
| 5 | + | 2968 | 3999 | ATG | TAA | 1032 | 343 | 1 | Putative capsid protein | putative capsid protein [Acinetobacter phage vB_AbaP_PD-AB9] | 100 | 100 | 0 | 5.41 | 38394.09 | YP_009189838.1 |
| 6 | + | 4055 | 4240 | ATG | TAA | 186 | 61 | 1 | hypothetical protein | hypothetical protein vBAbaPPDAB9_14 [Acinetobacter phage vB_AbaP_PD-AB9] | 100 | 100 | 2E-33 | 4.73 | 6458.33 | YP_009189837.1 |
| 7 | + | 4252 | 4545 | ATG | TAA | 294 | 97 | 1 | hypothetical protein | chromosome segregation ATPase-like protein [Acinetobacter phage vB_AbaP_B5] | 100 | 97 | 4E-55 | 5.13 | 9696.98 | ASN73449.1 |
| 8 | + | 4667 | 5296 | ATG | TAG | 630 | 209 | 1 | Putative tail tubular protein A | putative tail tubular protein A [Acinetobacter phage vB_AbaP_PD-AB9] | 100 | 100 | 4E-154 | 9.47 | 24420.15 | YP_009189835.1 |
| 9 | + | 5305 | 7596 | ATG | TAA | 2292 | 763 | 1 | Putative tail tubular protein B | putative tail tubular protein B [Acinetobacter phage vB_AbaP_PD-AB9] | 100 | 99 | 0 | 4.9 | 84463.87 | YP_009189834.1 |
| 10 | + | 7596 | 8267 | ATG | TAA | 672 | 223 | 1 | Putative internal virion protein B | hypothetical protein vBAbaPPDAB9_10 [Acinetobacter phage vB_AbaP_PD-AB9] | 100 | 100 | 2E-159 | 7.89 | 23491.11 | YP_009189833.1 |
| 11 | + | 8280 | 11165 | ATG | TAG | 2886 | 961 | 1 | Structural protein | structural protein [Acinetobacter phage vB_AbaP_B3] | 100 | 99 | 0 | 7.23 | 105800.7 | ASN73399.1 |
| 12 | + | 11175 | 14273 | ATG | TGA | 3099 | 1032 | 1 | Putative internal virion core protein | internal virion core protein [Acinetobacter phage IME200] | 100 | 99 | 0 | 5.88 | 114020.17 | YP_009216488.1 |
| 13 | + | 14279 | 16729 | ATG | TAA | 2451 | 816 | 1 | hypothetical protein | hypothetical protein [Acinetobacter baumannii] | 71 | 54 | 0 | 4.82 | 89813.95 | WP_115596580.1 |
| 14 | + | 16732 | 17475 | ATG | TAA | 744 | 247 | 1 | hypothetical protein | hypothetical protein [Acinetobacter baumannii] | 100 | 34 | 1E-29 | 7.86 | 26576.09 | WP_000192604.1 |
| 15 | + | 17485 | 17820 | ATG | TAA | 336 | 111 | 1 | Putative holin | putative holin [Acinetobacter phage Fri1] | 100 | 93 | 5E-68 | 4.87 | 11957.96 | YP_009203056.1 |
| 16 | + | 17807 | 18364 | ATG | TAG | 558 | 185 | 1 | Putative endolysin | endolysin [Acinetobacter phage IME200] | 100 | 98 | 4E-128 | 9.48 | 21017.43 | YP_009216491.1 |
| 17 | + | 18485 | 18793 | ATG | TAA | 309 | 102 | 1 | Putative DNA maturase | hypothetical protein Abp1_0051 [Acinetobacter phage Abp1] | 100 | 100 | 4E-67 | 4.47 | 11106.68 | YP_008058243.1 |
| 18 | + | 19733 | 20329 | ATG | TGA | 597 | 198 | 1 | Putative DNA maturase B | putative DNA maturase B [Acinetobacter phage SH-Ab 15519] | 100 | 99 | 0 | 6.32 | 72792.7 | APD19436.1 |
| 19 | + | 20831 | 21034 | ATG | TAG | 204 | 67 | 1 | hypothetical protein | hypothetical protein Abp1_0054 [Acinetobacter phage Abp1] | 100 | 100 | 1E-36 | 9.85 | 7020.29 | YP_008058246.1 |
| 20 | + | 22622 | 23131 | ATG | TAA | 510 | 169 | 1 | hypothetical protein | hypothetical protein vBAbaPPDAB9_48 [Acinetobacter phage vB_AbaP_PD-AB9] | 100 | 100 | 2E-120 | 6.96 | 18590.29 | YP_009189871.1 |
| 21 | + | 23133 | 23408 | ATG | TAA | 276 | 91 | 1 | hypothetical protein | hypothetical protein vBAbaPPDAB9_47 [Acinetobacter phage vB_AbaP_PD-AB9] | 100 | 100 | 4E-60 | 8.12 | 10197.61 | YP_009189870.1 |
| 22 | + | 23410 | 23784 | ATG | TAG | 375 | 124 | 1 | hypothetical protein | hypothetical protein vBAbaPPDAB9_46 [Acinetobacter phage vB_AbaP_PD-AB9] | 100 | 100 | 3E-87 | 4.74 | 14461.4 | YP_009189869.1 |
| 23 | + | 23850 | 24116 | TTG | TGA | 267 | 88 | 1 | hypothetical protein | hypothetical protein vBAbaPPDAB9_45 [Acinetobacter phage vB_AbaP_PD-AB9] | 95 | 100 | 3E-54 | 8.85 | 10638.06 | YP_009189868.1 |
| 24 | + | 24191 | 24787 | ATG | TGA | 597 | 198 | 1 | hypothetical protein | hypothetical protein vBAbaPPDAB9_44 [Acinetobacter phage vB_AbaP_PD-AB9] | 100 | 100 | 2E-147 | 6.15 | 22611.91 | YP_009189867.1 |
| 25 | + | 24875 | 25039 | ATG | TAG | 165 | 54 | 1 | hypothetical protein | hypothetical protein vBAbaPPDAB9_43 [Acinetobacter phage vB_AbaP_PD-AB9] | 100 | 100 | 3E-28 | 9.43 | 6339.87 | YP_009189866.1 |
| 26 | + | 25048 | 25446 | ATG | TAG | 399 | 132 | 1 | hypothetical protein | hypothetical protein vBAbaPPDAB9_42 [Acinetobacter phage vB_AbaP_PD-AB9] | 100 | 100 | 2E-93 | 6.96 | 14803.78 | YP_009189865.1 |
| 27 | + | 25468 | 26004 | ATG | TAA | 537 | 178 | 1 | hypothetical protein | hypothetical protein vBAbaPPDAB9_41 [Acinetobacter phage vB_AbaP_PD-AB9] | 100 | 100 | 3E-129 | 9.52 | 19842.8 | YP_009189864.1 |
| 28 | + | 26006 | 26440 | ATG | TAA | 435 | 144 | 1 | hypothetical protein | hypothetical protein vBAbaPPDAB9_40 [Acinetobacter phage vB_AbaP_PD-AB9] | 100 | 100 | 5E-103 | 9.32 | 16288.88 | YP_009189863.1 |
| 29 | + | 26451 | 26618 | ATG | TAA | 168 | 55 | 1 | hypothetical protein | hypothetical protein vBAbaPPDAB9_39 [Acinetobacter phage vB_AbaP_PD-AB9] | 100 | 100 | 3E-30 | 10.15 | 6826.76 | YP_009189862.1 |
| 30 | + | 26605 | 26796 | ATG | TGA | 192 | 63 | 1 | hypothetical protein | hypothetical protein Abp1_0013 [Acinetobacter phage Abp1] | 100 | 100 | 3E-39 | 9.83 | 7123.22 | YP_008058205.1 |
| 31 | + | 26793 | 27011 | ATG | TAA | 219 | 72 | 1 | hypothetical protein | hypothetical protein vBAbaPPDAB9_37 [Acinetobacter phage vB_AbaP_PD-AB9] | 100 | 100 | 1E-44 | 9.05 | 7887.03 | YP_009189860.1 |
| 32 | + | 27001 | 27210 | TTG | TAG | 210 | 69 | 1 | hypothetical protein | hypothetical protein Abp1_0015 [Acinetobacter phage Abp1] | 100 | 100 | 3E-43 | 8.98 | 7856.85 | YP_008058207.1 |
| 33 | + | 27233 | 27682 | ATG | TGA | 450 | 149 | 1 | hypothetical protein | hypothetical protein Abp1_0016 [Acinetobacter phage Abp1] | 100 | 99 | 1E-106 | 9.57 | 16931.54 | YP_008058208.1 |
| 34 | + | 27712 | 28467 | ATG | TAA | 756 | 251 | 1 | hypothetical protein | hypothetical protein vBAbaPPDAB9_34 [Acinetobacter phage vB_AbaP_PD-AB9] | 100 | 99 | 0 | 8.85 | 28817.26 | YP_009189857.1 |
| 35 | + | 28467 | 28784 | ATG | TAA | 318 | 105 | 1 | hypothetical protein | hypothetical protein vBAbaPPDAB9_33 [Acinetobacter phage vB_AbaP_PD-AB9] | 100 | 100 | 2E-70 | 4.8 | 11813.96 | YP_009189856.1 |
| 36 | + | 28784 | 29017 | ATG | TAA | 234 | 77 | 1 | hypothetical protein | hypothetical protein vBAbaPPDAB9_32 [Acinetobacter phage vB_AbaP_PD-AB9] | 100 | 100 | 1E-50 | 6.81 | 8802.19 | YP_009189855.1 |
| 37 | + | 29030 | 30328 | ATG | TGA | 1299 | 432 | 1 | Putative DNA helicase | putative DNA helicase [Acinetobacter phage vB_AbaP_PD-AB9] | 100 | 100 | 0 | 5.44 | 48336.47 | YP_009189854.1 |
| 38 | + | 30325 | 31368 | ATG | TGA | 1044 | 347 | 1 | hypothetical protein | hypothetical protein vBAbaPPDAB9_30 [Acinetobacter phage vB_AbaP_PD-AB9] | 100 | 100 | 0 | 6.81 | 39189.79 | YP_009189853.1 |
| 39 | + | 31671 | 33971 | ATG | TAG | 2301 | 766 | 1 | Putative DNA polymerase | putative DNA polymerase [Acinetobacter phage vB_AbaP_PD-AB9] | 100 | 100 | 0 | 5.59 | 87309.14 | YP_009189852.1 |
| 40 | + | 33980 | 34459 | ATG | TAA | 480 | 159 | 1 | Putative HNH endonuclease | putative HNH endonuclease [Acinetobacter phage vB_AbaP_PD-AB9] | 100 | 100 | 1E-116 | 9.92 | 18575.27 | YP_009189851.1 |
| 41 | + | 34477 | 35367 | ATG | TAA | 891 | 296 | 1 | hypothetical protein | hypothetical protein vBAbaPPDAB9_27 [Acinetobacter phage vB_AbaP_PD-AB9] | 100 | 100 | 0 | 5.02 | 32368.55 | YP_009189850.1 |
| 42 | + | 35576 | 36532 | ATG | TGA | 957 | 318 | 1 | Putative DNA exonuclease | putative DNA exonuclease [Acinetobacter phage vB_AbaP_PD-AB9] | 100 | 100 | 0 | 5.79 | 36223.16 | YP_009189849.1 |
| 43 | + | 36513 | 36950 | ATG | TGA | 438 | 145 | 1 | Phage-associated homing endonuclease | homing endonuclease [Acinetobacter phage vB_AbaP_PD-AB9] | 100 | 99 | 8E-104 | 9.73 | 16549.07 | YP_009189848.1 |
| 44 | + | 36947 | 37387 | ATG | TAA | 441 | 146 | 1 | Putative DNA endonuclease VII | putative DNA endonuclease VII [Acinetobacter phage vB_AbaP_PD-AB9] | 100 | 100 | 2E-105 | 9.77 | 16597.02 | YP_009189847.1 |
| 45 | + | 37391 | 38326 | ATG | TAA | 936 | 311 | 1 | hypothetical protein | hypothetical protein vBAbaPPDAB9_23 [Acinetobacter phage vB_AbaP_PD-AB9] | 100 | 100 | 0 | 7.76 | 35461.66 | YP_009189846.1 |
| 46 | + | 38326 | 38457 | ATG | TGA | 132 | 43 | 1 | hypothetical protein | hypothetical protein vBAbaPPDAB9_22 [Acinetobacter phage vB_AbaP_PD-AB9] | 100 | 100 | 1E-24 | 9.27 | 5282.27 | YP_009189845.1 |
| 47 | + | 38454 | 39098 | ATG | TAG | 645 | 214 | 1 | Putative dNMP kinase | putative dNMP kinase [Acinetobacter phage vB_AbaP_PD-AB9] | 100 | 100 | 1E-154 | 4.96 | 24554.9 | YP_009189844.1 |
| 48 | + | 39107 | 41524 | ATG | TAA | 2418 | 805 | 1 | DNA-directed RNA polymerase | DNA-directed RNA polymerase [Acinetobacter phage vB_AbaP_PD-AB9] | 100 | 100 | 0 | 6.35 | 90771 | YP_009189843.1 |

Figure 1. Biological Characterization of phage PD6A3.


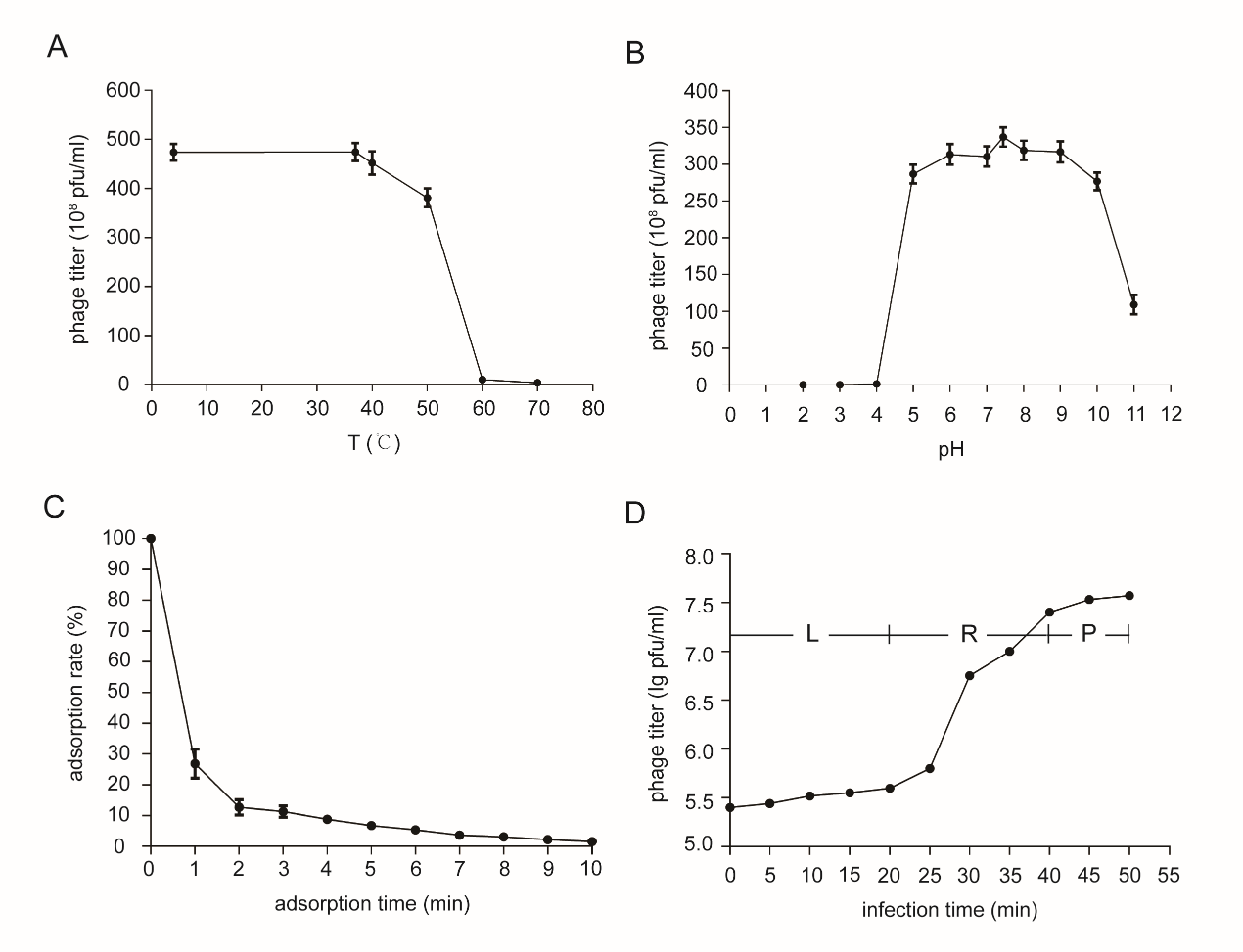


(A)Temperature stability curves of phage PD-6A3. Phage was incubated at different temperatures values for one hour before determining the phage titer. (B)Ph stability curves of phage PD-6A3. Phage was incubated at different pH values for one hour before determining the phage titer. (C)Adsorption curve of phage PD-6A3. Aliquots were taken at intervals of one minute (up to 10 min). And then the filtrates containing the non-adsorbed phages were titrated to calculate the adsorption rates. (E) One-step growth curve of phage PD-6A3. Latent time and burst size of phage PD-6A3 were indicated in the triphasic curve. L: latent phase; R: rise phase; P: plateau phase.

PD-6A3: A novel Acinetobacter baumannii Phage.

pH: hydrogen ion concentration

Figure 2. The minimum lethal dose of mice sepsis(pfu/ml).


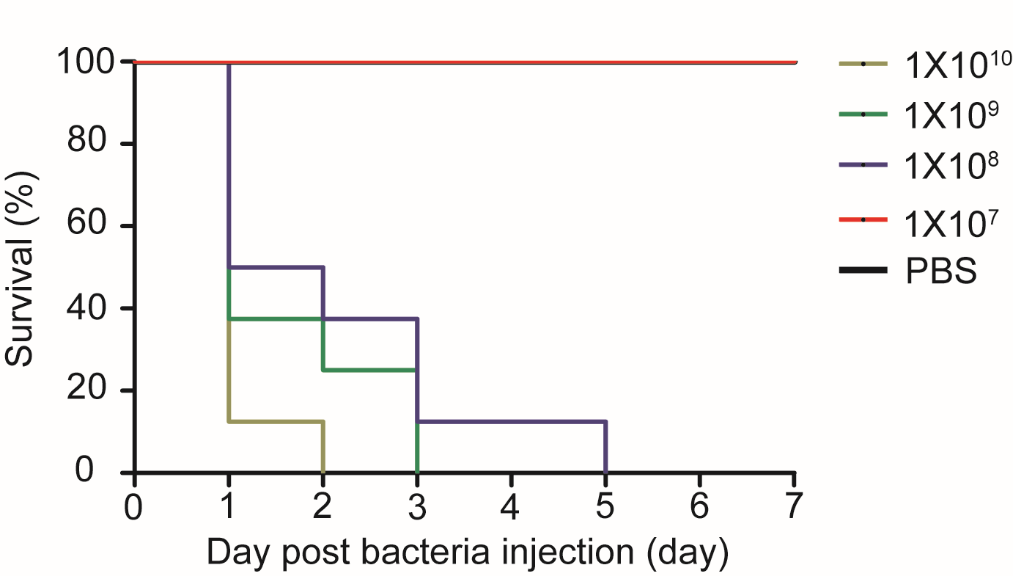

Supplement: Supplementary file 1 [file Data_Sheet_1.doc]
